# Ischemic postconditioning protects nonculprit coronary arteries against ischemia-reperfusion injury via downregulating miR-92a, miR-328 and miR-494

**DOI:** 10.18632/aging.203971

**Published:** 2022-03-23

**Authors:** Jian Wang, Wu Wang, Chengying Yan, Tianzhen Wang

**Affiliations:** 1 Department of Cardiology, Beijing Geriatric Hospital, Beijing 100095, Beijing, China; 2 Department of Cardiology, Xining First People’s Hospital, Xining 810001, Qinghai, China; 3 Hengduan House, RDFZ Chaoyang Branch School, Beijing 100028, Beijing, China

**Keywords:** nonculprit lesions, ischemic postconditioning, ischemia-reperfusion, miRNAs, mRNAs

## Abstract

Background: Nonculprit lesions are closely related to the prognosis of patients with ST-segment elevation myocardial infarction (STEMI). Our previous research found that ischemic postconditioning (IP) could inhibit the progression of nonculprit lesions. However, the mechanism by which IP regulates the occurrence and development of nonculprit lesions remains unclear.

Methods: Firstly, a rabbit ischemia-reperfusion (IR) model was constructed. Next, the morphological characteristics of the coronary arterial tissues and myocardial tissues of the rabbits were observed using hematoxylin-eosin (H&E) staining. Then, western blot was performed to detect the expressions of AT1, Cx43, β-tubulin, Bax, Bcl-2 and cleaved caspase 3. Finally, to further confirm the effect of IP on nonculprit coronary arterial tissues, an *in vitro* model of oxygen and glucose deprivation/reperfusion (OGD/R) was established.

Results: IR notably induced the cells apoptosis in nonculprit coronary arterial tissues and in myocardial tissues, while IR-induced cell apoptosis was significantly inhibited by IP. In addition, IP protected nonculprit coronary arterial tissues against IR via downregulating miR-92a, miR-328 and miR-494 and mRNA AT1, Cx43 and β-tubulin. Consistently, OGD/R-induced injury of Human umbilical vein endothelial cells (HUVECs) was reversed by IP.

Conclusions: In this study, IP could protect nonculprit coronary arteries against IR injury via downregulating miR-92a, miR-328 and miR-494. Our findings may provide new directions for the treatment of nonculprit lesions.

## INTRODUCTION

Myocardial infarction (MI) is the leading cause of mortality globally due to the death of cardiomyocytes [1]. ST-segment elevation myocardial infarction (STEMI) is a type of MI [2]. To some extent, STEMI can directly threaten the life of patients [3]. Currently, the fundamental treatment strategy of STEMI is the earliest restoration of myocardial perfusion [4]. And the most common and effective treatment for STEMI is primary percutaneous coronary intervention (PPCI), with a high success rate of blood flow [5, 6]. PPCI can greatly improve the clinical prognosis of patients with STEMI [7]. However, the patients with STEMI are more likely to have a three-vessel lesion [7]. Moreover, the prognosis of patients with a three-vessel lesion is still less than ideal [8]. Recent studies have found that the presence of nonculprit lesions may be closely related to the prognosis of patients with STEMI [9]. In addition, Thim et al. reported that nonculprit lesions are often found in patients with STEMI [10].

Ischemic postconditioning (IP) has become a hot spot in the study of anti-ischemia/reperfusion injury all around the world [11]. IP refers to repeated, transient reperfusion/ischemia treatment or drug intervention prior to long reperfusion after tissue or organ ischemia [12]. In addition, IP can reduce tissue reperfusion injury by mobilizing endogenous repair mechanism [12]. Our previous research found that IP could inhibit the progression of nonculprit lesions [13]. However, the mechanism by which IP regulated the occurrence and development of nonculprit lesions remains unclear.

MicroRNAs (miRNAs) are short sequences of RNA that do not code for proteins in eukaryotes [14]. MiRNAs are highly tissue specific and can determine the functional specificity of cells [15]. These results indicate that miRNAs play a variety of roles in the regulation of cell growth and development [16]. For example, Gao et al. indicated that IP decreased the apoptosis of cardiomyocytes in patients undergoing cardiac surgery by regulating miR-1 and miR-21 [17]. In addition, Varga et al. reported that IP significantly affected the expressions of miR-328 and miR-208 and exhibited myocardial protection effect in an ischemia-reperfusion (IR) rat model [18].

In this study, we aim to explore the effects of IP on miRNAs expression in nonculprit coronary arteries of rabbits. Our finding illustrated that IP protected nonculprit coronary arteries against IR-induced injury via downregulating miR-92a, miR-328 and miR-494. This study might provide hopeful therapeutic strategy and theoretical support for the treatment of nonculprit lesions.

## RESULTS

### IP protected nonculprit coronary arteries against IR injury

**Figure 1:**
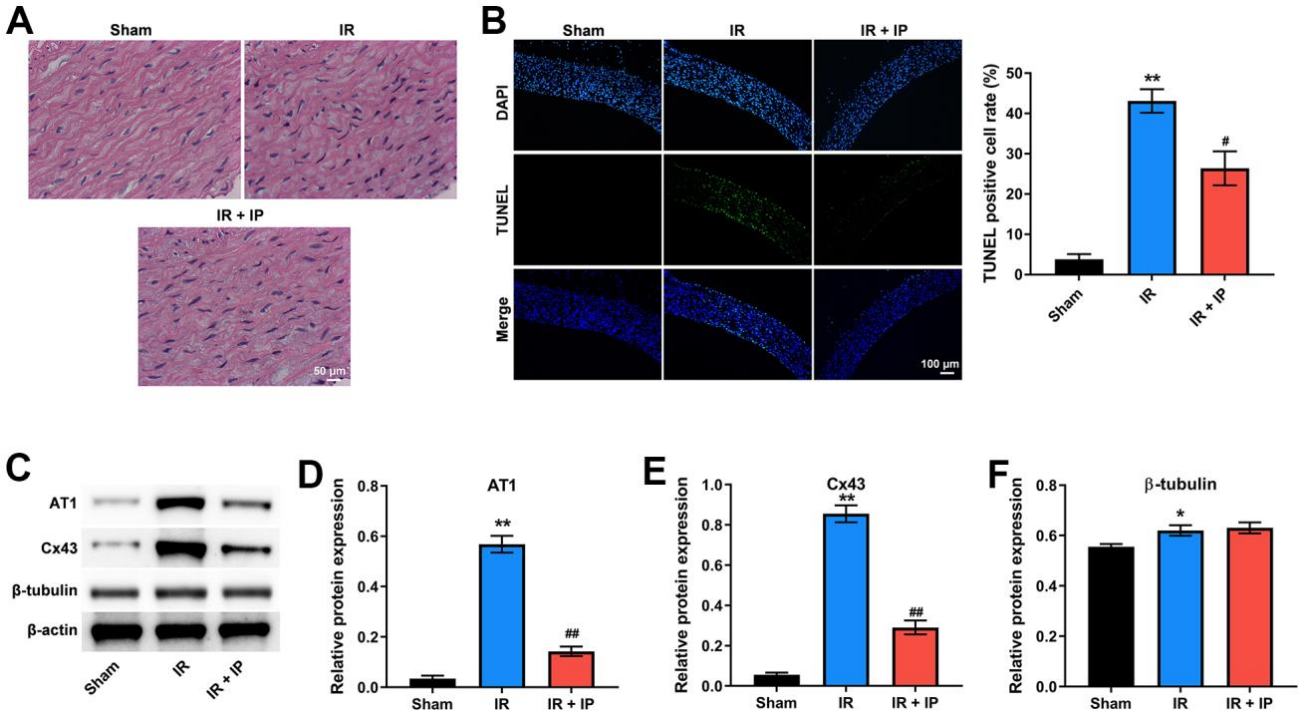
**IP protected nonculprit coronary arteries against IR injury.** (**A**) H&E staining assay was conducted to observe the morphology of nonculprit coronary arterial tissues. (**B**) TUNEL and DAPI staining assay was performed to detect the apoptosis of nonculprit coronary arterial tissues. (**C**–**F**) Western blot assay was used to detect the expressions of AT1, Cx43 and β-tubulin. *P<0.01, **P<0.01 compared with the sham group; ^#^P<0.01, ^##^P<0.01 compared with the IR group. IP, ischemic postconditioning; IR, ischemia-reperfusion.

To investigate the effect of IP on the morphology of nonculprit coronary arterial tissues, H&E staining assay was conducted. As shown in Figure 1A, the structure of nonculprit coronary arterial tissues was clear and orderly in sham group. Interestingly, the morphology of cells in the IR or IR+IP treated group was not markedly different from that in the sham group. In addition, IR notably induced the cells apoptosis of nonculprit coronary arterial tissues compared with the sham group, while IR-induced apoptosis was significantly inhibited by IP (Figure 1B).

Our previous studies have shown that IP could significantly affect the expression of mRNA angiotensin II receptor type 1 (AT1), connexin 43 (Cx43), and β-tubulin, which are key factors in regulating the state of blood vessels and play an important role in nonculprit lesions in the patients with MI [13]. Consistently, western blot assay showed that IR obviously upregulated the level of AT1, Cx43 and β-tubulin in nonculprit coronary arterial tissues; however, these increases of AT1 and Cx43 were notably alleviated by IP. And IP had no significant effect on the increase of β-tubulin induced by IR (Figure 1C–1F). Taken together, IP significantly protected nonculprit coronary arteries against IR injury.

### Effect of IP on miRNAs expression in nonculprit coronary arterial tissues

**Figure 2:**
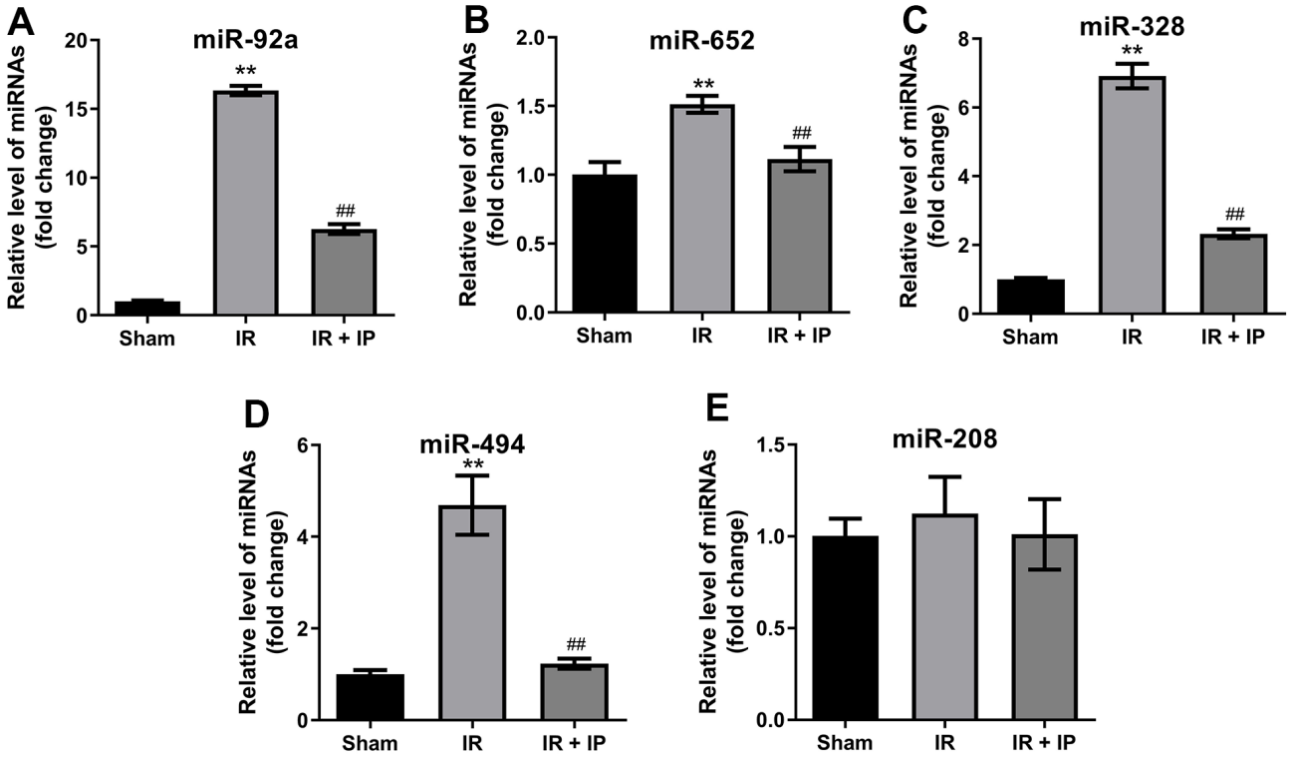
**Effect of IP on miRNAs expression in nonculprit coronary arterial tissues.** (**A**–**E**) RT-qPCR was performed to detect the expression of miR-92a, miR-652, miR-328, miR-494 and miR-208 in nonculprit coronary arterial tissues. **P<0.01 compared with the sham group; ^##^P<0.01 compared with the IR group.

Previous studies reported that miR-92a, miR-652, miR-328, miR-494 and miR-208 are closely related to IR and non-infarction-related arterial diseases [18]. Thereby, we explored the effect of IP on these miRNAs expression in nonculprit coronary arterial tissues. RT-qPCR results showed that IR significantly promoted the expression of miR-92a, miR-652, miR-328, miR-494 and miR-208 in nonculprit coronary arterial tissues, while IP notably reversed these phenomena (Figure 2A–2E).

### IP protected myocardial tissues against IR injury

**Figure 3:**
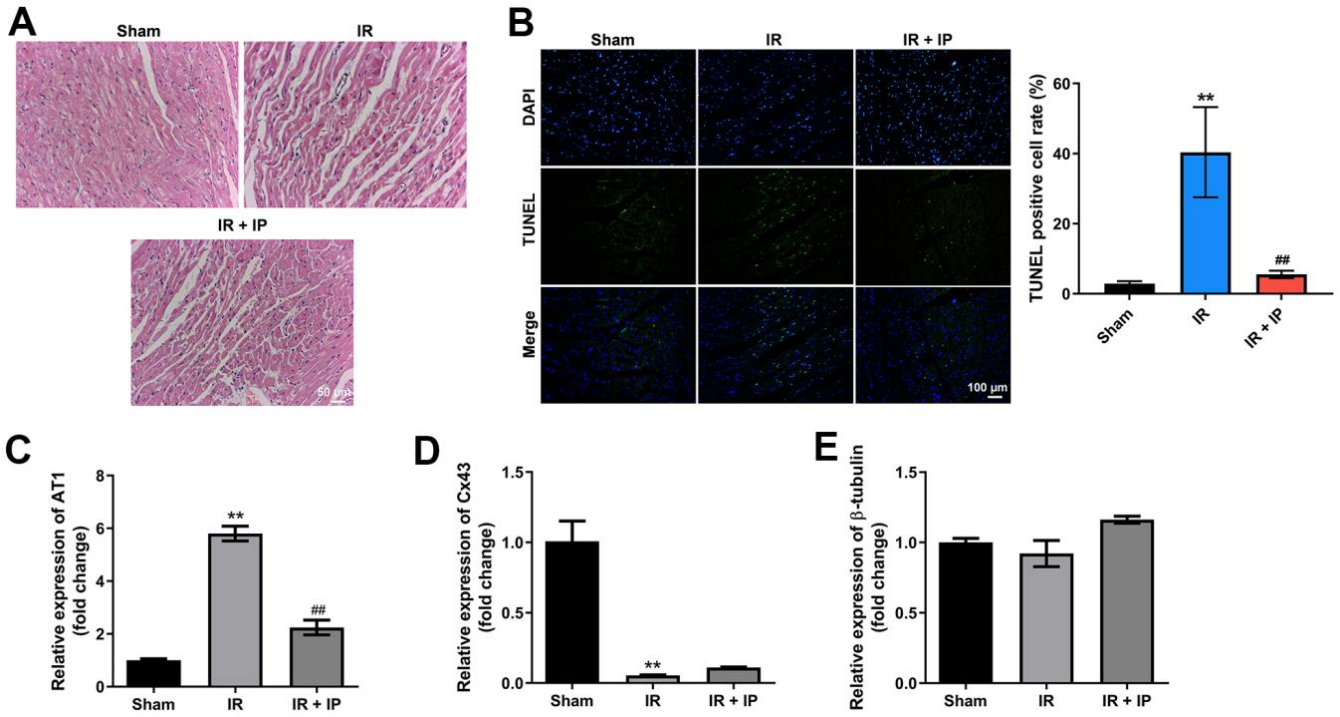
**IP protected myocardial tissues against IR injury.** (**A**) H&E staining assay was conducted to observe the morphology of nonculprit myocardial tissues. (**B**) TUNEL and DAPI staining assay was performed to detect the apoptosis of nonculprit myocardial tissues. (**C**–**E**) RT-qPCR assay was used to detect the expression of AT1, Cx43 and β-tubulin. **P<0.01 compared with the sham group; ^##^P<0.01 compared with the IR group.

Next, the effect of IP on the morphology of myocardial tissues was investigated. The results of H&E staining showed that the myocardial tissues were intact in the sham group, while the myocardial tissues were disordered with obvious fractures in the IR group. However, IP notably reversed IR induced-pathological lesions (Figure 3A). Meanwhile, IR notably induced the cell apoptosis of myocardial tissues, which was reversed by IP (Figure 3B). In addition, RT-qPCR results indicated that IR significantly increased the level of AT1 and decreased the expressions of Cx43 and β-tubulin in myocardial tissues, while these effects were all reversed by IP (Figure 3C–3E). Thus, IP could significantly protect myocardial tissues against IR injury.

### Effect of IP on miRNAs expression in myocardial tissues

**Figure 4:**
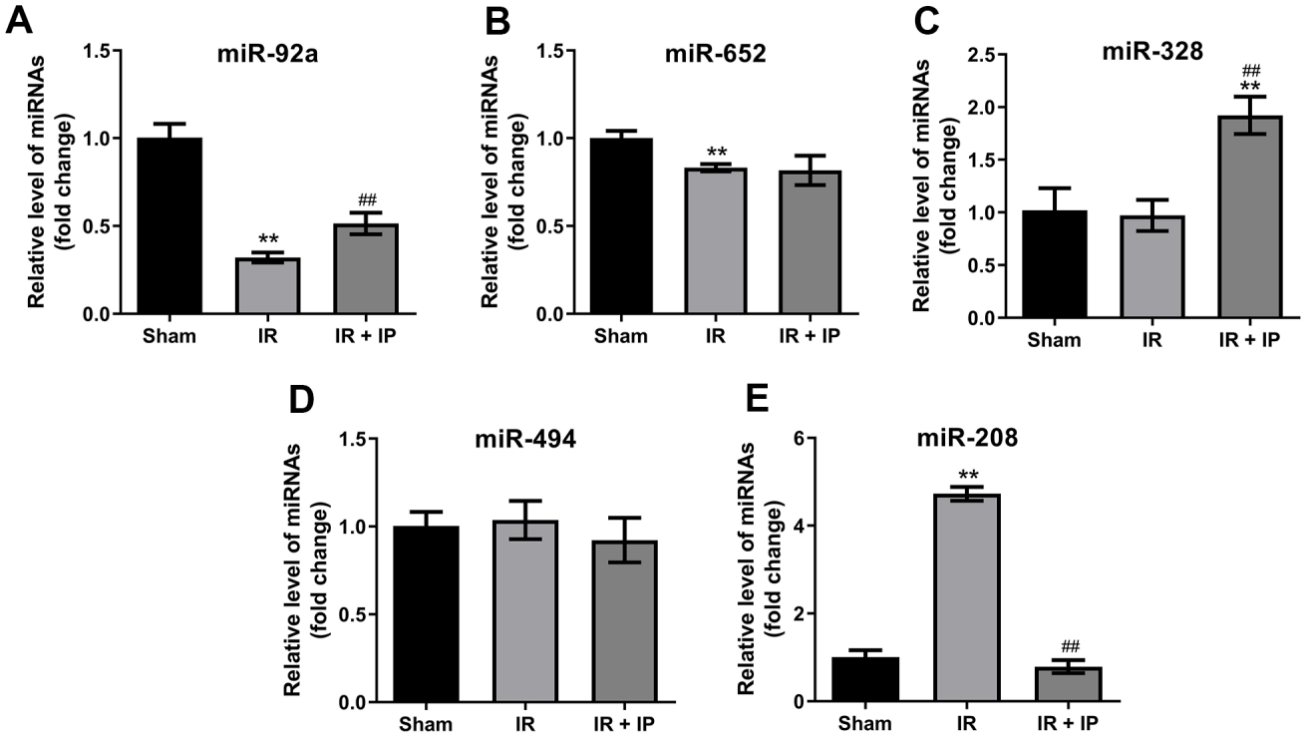
**Effect of IP on miRNAs expression in myocardial tissues.** (**A**–**E**) RT-qPCR was performed to detect the expression of miR-92a, miR-652, miR-328, miR-494 and miR-208 in nonculprit myocardial tissues. **P<0.01 compared with the sham group; ^##^P<0.01 compared with the IR group.

We next investigate the effect of IP on miRNAs expression in myocardial tissues. As indicated in RT-qPCR results IR remarkably downregulated the levels of miR-92a and miR-652 in myocardial tissues. In contrast, IP significantly upregulated the level of miR-92a in myocardial tissues that were treated with IR; but it had no effect on the expression of miR-652 (Figure 4A, 4B). In addition, IR did not affect the expression of miR-328 and miR-494 in myocardial tissues. Interesting, IP significantly increased the level of miR-328; but it had no effect on miR-494 expression (Figure 4C, 4D). Importantly, IR significantly increased the expression of miR-208, and this increase was markedly reversed by IP (Figure 4E).

### IP protected human umbilical vein endothelial cells (HUVECs) against OGD/R via downregulating miR-92a, miR-328 and miR-494

**Figure 5:**
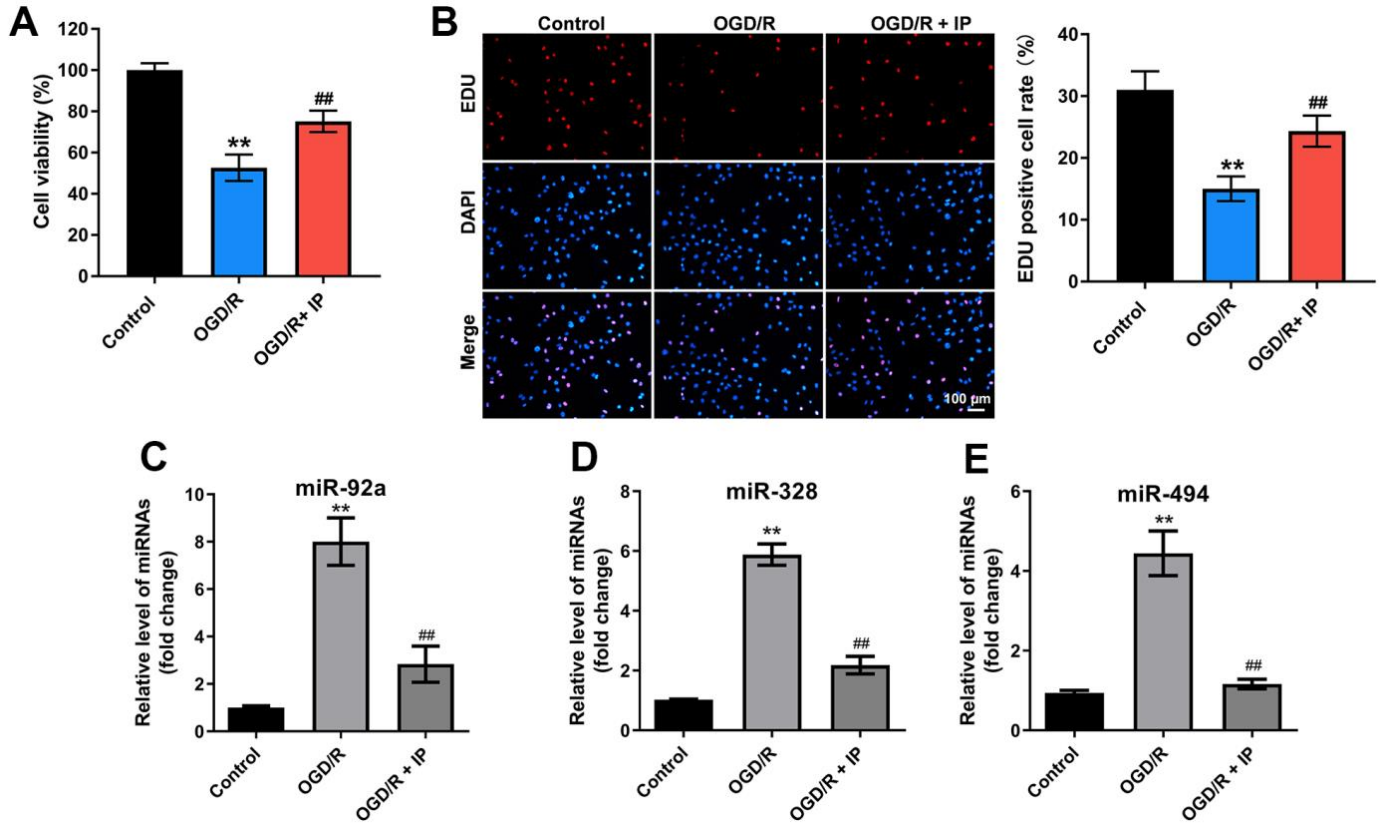
**IP protected HUVECs against OGD/R via downregulating miR-92a, miR-328 and miR-494.** To construct a model of OGD/R *in vitro*, HUVECs were cultured in glucose-free and serum-free DMEM at 95% N_2_ and 5% CO_2_ for 4 h. Then, HUVECs were cultured in DMEM containing 10% serum at 95% N_2_ and 5% CO_2_ for 18 h. (**A**) CCK-8 staining assay was used to detect the viability of HUVECs. (**B**) EdU staining assay was used to detect the proliferation of HUVECs. (**C**–**E**) RT-qPCR was performed to detect the expression of miR-92a, miR-328 and miR-494 in HUVECs. **P<0.01 compared with the sham group; ^##^P<0.01 compared with the IR group.

To further confirm the effect of IP on nonculprit coronary arterial tissues, an *in vitro* model of OGD/R was established. The results of CCK-8 and EdU staining showed OGD/R significantly inhibited the viability and proliferation of HUVECs; however, OGD/R-induced cytotoxicity was significantly reversed by IP (Figure 5A, 5B). In addition, OGD/R effectively upregulated the expression of miR-92a, miR-328 and miR-494 in HUVECs; however, these phenomena were notably reversed by IP as well (Figure 5C–5E). All in all, these data suggested that IP protected HUVECs against OGD/R via downregulating miR-92a, miR-328 and miR-494.

### IP inhibited OGD/R-induced apoptosis of HUVECs via downregulating mRNA AT1, Cx43 and β-tubulin

**Figure 6:**
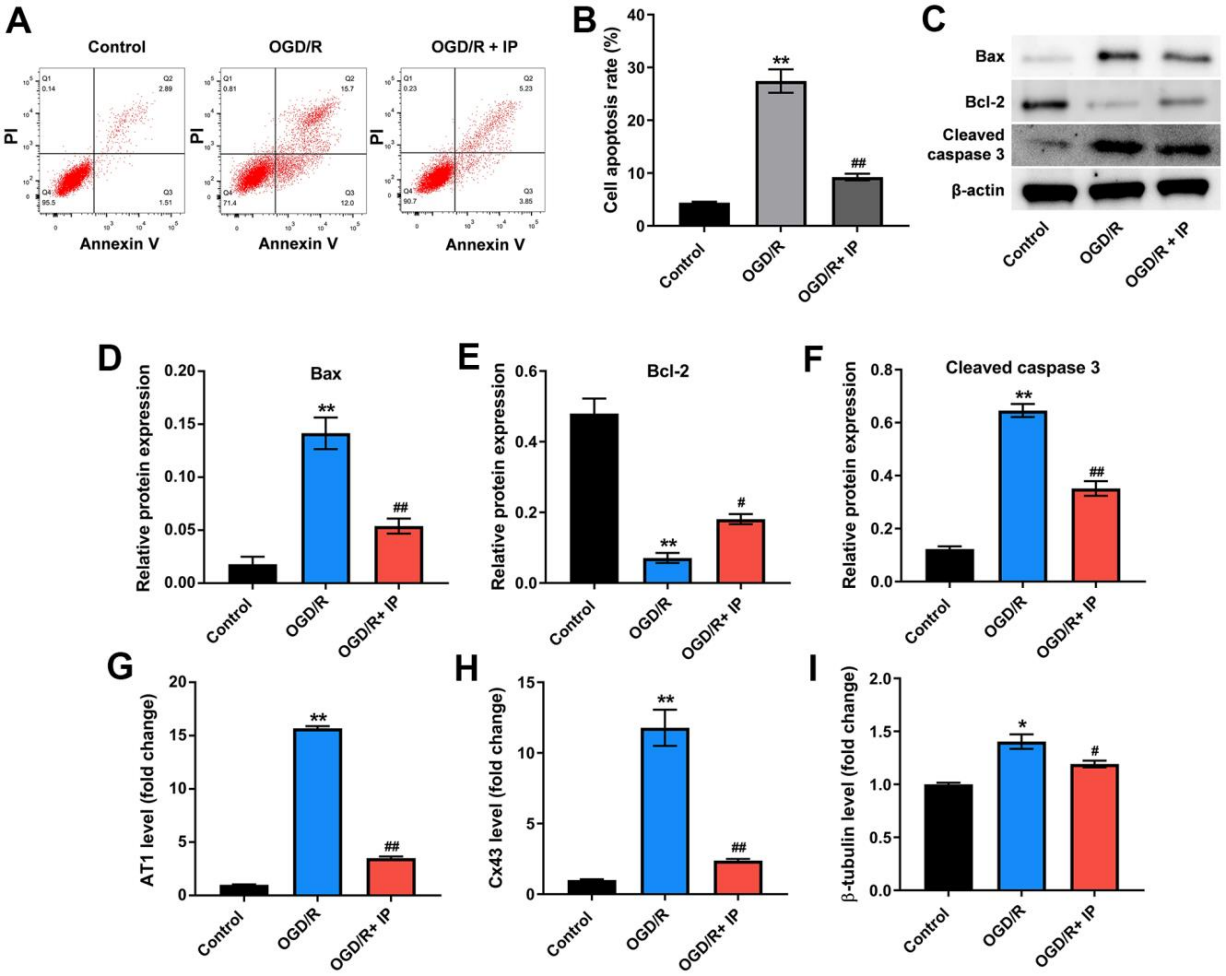
**IP inhibited OGD/R-induced apoptosis of HUVECs via downregulating mRNA AT1, Cx43 and β-tubulin.** (**A**, **B**) Flow cytometric analysis was performed to detect the apoptosis of HUVECs. (**C**–**F**) Western blot assay was used to detect the expression of Bax, Bcl-2 and cleaved caspase 3 in HUVECs. (**G**–**I**) RT-qPCR was performed to detect the expression of AT1, Cx43 and β-tubulin. *P<0.01, **P<0.01 compared with the sham group; ^#^P<0.01, ^##^P<0.01 compared with the IR group.

Finally, to investigate the effect of IP on the apoptosis of HUVECs that were treated with OGD/R, flow cytometric analysis was performed. As shown in Figure 6A, 6B, OGD/R significantly induced the apoptosis of HUVECs, while IP markedly reversed OGD/R-induced apoptosis. Consistent with these data, western blot assay showed that OGD/R remarkably increased the level of Bax and cleaved caspase 3 and decreased the level of Bcl-2 in HUVECs, while these phenomena were all reversed by IP (Figure 6C–6F). Moreover, OGD/R largely increased the expressions of mRNA AT1, Cx43 and β-tubulin in HUVECs; however, these changes were abrogated by IP as well (Figure 6G–6I). All these data suggested that IP inhibited OGD/R-induced apoptosis of HUVECs via downregulating mRNA AT1, Cx43 and β-tubulin.

## DISCUSSION

In recent years, the mortality of acute myocardial infarction (AMI) has increased significantly. PPCI targeting criminal arteries of AMI could notably reduce the mortality of patients with AMI [19]. However, the patients with STEMI are more likely to have a three-vessel lesion [7]. In other words, there is a higher probability of finding nonculprit lesions in the patients with STEMI [10].

There are few studies on the progression of nonculprit arteries disease in AMI. Our previous studies reported that the influence of IP on cell apoptosis in nonculprit coronary arteries [18]. In addition, Gibson et al. reported that the perfusion of criminal arteries and nonculprit arteries was impaired in acute myocardial infarction [20]. PPCI could relieve the occlusion of criminal arteries, and the blood perfusion of nonculprit arteries could be improved as well [20]. In this study, these results showed that OGD/R significantly inhibited the viability and proliferation of HUVECs; however, OGD/R-induced cytotoxicity was significantly reversed by IP. Our present study was consistent with previous studies suggesting that IP might be a good treatment for nonculprit lesions in the patients with STEMI.

Interestingly, these effects of IR+IP on the morphology of nonculprit coronary arterial tissues and myocardial tissues were different. The morphology of cells in the IR or IR+IP treated group was not remarkably different from that in the sham group. However, the nonculprit myocardial tissues were disordered with obvious fractures in the IR group, while IP notably reversed IR induced-lesion. In addition, these effects of IR+IP on miR-92a, miR-652, miR-328, miR-494, miR-208 and mRNA AT1, Cx43 and β-tubulin in nonculprit coronary arterial tissues or in myocardial tissues were different. Moreover, Varga et al. showed that miR-92a, miR-652, miR-328, miR-494 and miR-208 were closely related to IR and non-infarction-related arterial diseases [18]. In the current study, these miRNAs were extensively involved in nonculprit coronary arterial tissues and myocardial tissues that were treated with IR or IR+IP. In addition, it was confirmed that IP protected HUVECs against OGD/R via downregulating miR-92a, miR-328 and miR-494 *in vitro*. Our study did not conflict with previous research. All these results commonly suggest that miR-92a, miR-652, miR-328, miR-494 and miR-208 could be used as new biomarkers in nonculprit lesions.

Indeed, there are some limitations in the current study. For example, the related functions of miR-92a, miR-652, miR-328, miR-494 and miR-208 should be further investigated. In addition, the mechanism by which IP regulates the development of nonculprit lesions should be further explored.

In conclusion, IP could protect nonculprit coronary arteries against IR injury via downregulating miR-92a, miR-328 and miR-494. This finding showed that IP might provide new directions for the treatment of nonculprit lesions in the patients with STEMI.

## MATERIALS AND METHODS

### Hyperlipidemia animal model

All animal procedures were approved by the Ethics Committee of Xining First People’s Hospital. National Institutes of Health Guide for the Care and Use of Laboratory Animals was followed strictly. Healthy male rabbits were purchased from Vital River (Beijing, China). Firstly, these rabbits (n = 40) were randomized into two groups: the hyperlipidemia group (n = 30) and the control group (n = 10). Rabbits were fed a high-fat diet (the hyperlipidemia group) or a normal diet (the control group) for 11 weeks plus 3 days. Later on, the rabbits were fasting for 12 hours. Next, ear venous blood was taken and followed by serum total cholesterol (TC) was measured. The serum TC content of rabbits in the control group was 1-2 mmol/L. Rabbits that serum TC content was 3 times than that in the control group were selected for myocardial ischemia reperfusion modeling.

### Myocardial ischemia-reperfusion (IR) animal model

The acute myocardial ischemia animal model was constructed according to our previous published literature [13].

### Cell culture

HUVECs were obtained from ATCC (Manassas, VA, USA). Cells were cultured in DMEM (Thermo, Waltham, MA, USA). DMEM contained 10% FBS and 100 U/mL penicillin and streptomycin in 5% CO_2_, at 37° C. To construct a model of oxygen and glucose deprivation/reperfusion (OGD/R) *in vitro*, HUVECs were cultured in glucose-free and serum-free DMEM at 95% N_2_ and 5% CO_2_ for 4 h. Then, HUVECs were cultured in DMEM containing 10% serum at 95% N_2_ and 5% CO_2_ for 18 h.

### Hematoxylin-eosin (H&E) stain and observation

The coronary arterial tissues and myocardial tissues of the rabbits were separated carefully and followed by embedded in paraffin. Later on, the tissues were stained with H&E staining. Then, a microscope was used to observe stained cells.

### Terminal deoxynucleotidyl transferase (TdT)-mediated dUTP nick end labeling (TUNEL) assay

The rate of TUNEL positive cells in nonculprit coronary arterial tissues and myocardial tissues were detected by TUNEL and 4’, 6-diamidino-2-phenylindole (DAPI) dyes. Firstly, tissues were embedded in paraffin. Next, TUNEL reaction solution was added to the samples and incubated at 37° C for 60 min. Then, the samples were rinsed with PBS for 3 times, 5 min each time. Later on, DAPI was added to the sample and incubated in the dark for 5 min. Finally, a fluorescence microscope was used to observe the results of staining.

### Reverse transcription-quantitative polymerase chain reaction (RT-qPCR)

TRIzol (ELK Biotechnology, Wuhan, China) were used to extract total RNA from cells. Later on, complimentary DNA (cDNA) was synthesized from these extracted RNA using EntiLink™ 1st Strand cDNA Synthesis Kit (ELK Biotechnology). RT-qPCR was performed using EnTurbo™ SYBR Green PCR SuperMix Kit (ELK Biotechnology) and run on the StepOne™ Real-Time PCR (Life Technologies, Carlsbad, CA, USA).

### Western blot assay

Western blot assay was performed to detect the expressions of AT1, Cx43, β-tubulin, Bax, Bcl-2 and cleaved caspase 3 according to the manufacturer’s instructions. Protein lysis buffer (Beyotime, Shanghai, China) were used to extract total protein from nonculprit coronary arterial tissues or HUVECs. To determine the concentration of protein, BCA kit (Nanjing Jiancheng Bioengineering Institute Nanjing, China) was performed. Next, 30 μg/lane proteins were separated using 10 % SDS-PAGE and followed by transferred to PVDF membranes (Millipore, Billerica, MA, USA). Then, 5% skim milk was used to block the PVDF membranes at room temperature for 1 h. After that, the PVDF membranes were incubated with primary antibodies at 4° C overnight. The primary antibodies used in this study were as follows: anti-β-actin (1:1000, Abcam, Cambridge, MA, USA, cat. no. ab8226), anti-AT1 (1:1000, Abcam, cat. no. ab124734), anti-Cx43 (1:1000, Abcam, cat. no. ab11370), anti-β-tubulin (1:1000, Abcam, cat. no. ab179513), anti-Bax (1:1000, Abcam, cat. no. ab32503), anti-Bcl-2 (1:1000, Abcam, cat. no. ab196495), anti-cleaved caspase 3 (1:1000, Abcam, cat. no. ab2302). Protein expression levels were standardized against β-actin. Later on, the PVDF membranes were incubated with horseradish peroxidase-conjugated secondary antibodies (Goat anti-rabbit IgG antibody (1:5000, Abcam, cat. no. ab6721) for 1 h at room temperature. Subsequently, efficient chemiluminescence (ECL) kit (Thermo) was used to visualize the protein.

### CCK-8 assay

HUVECs were seeded in 96-well plates overnight at a density of 5x10^3^ cells per well. Next, HUVECs were treated with OGD/R or OGD/R+IP. Then, HUVECs were treated with 10 μL CCK-8 reagent (Dojindo, Kumamoto, Japan) and incubated at 37° C for 2 h. Finally, a microplate reader (Bio-Rad, Hercules, CA, USA) was performed to measure the absorbance of HUVECs at 450 nm.

### EdU (5-Ethynyl-2’-deoxyuridine) staining assay

Cell-Light EdU Apollo567 *In Vitro* Kit (100T) (C10310-1) was provided by Guangzhou RiboBio Co., Ltd. (Guangzhou, Guangdong, China). This kit was performed to detect cell proliferation. Firstly, HUVECs (4x10^3^ cells per well) were seeded onto 96-well plates. After that, the cells were cultured with 50 μM EdU for 2 h followed by washed using PBS. Then, cells were incubated with 100 μL PBS containing 4% paraformaldehyde for 30 min. After that, cells were incubated with 100 μL 1 mg/mL DAPI for 10 min at room temperature in the darkness. Finally, a fluorescence microscope was used to observe the results of staining.

### Flow cytometric analysis

Annexin V-FITC Apoptosis Detection Kit (C1062S) was purchased from Beyotime (Shanghai, China). This kit was performed to detect the apoptosis of HUVECs. HUVECs were seeded in 6-well plates overnight at a density of 5x10^4^/mL. Next, HUVECs were treated with OGD/R or OGD/R+IP. After that, cells were stained with 5 μL annexin V and PI in the dark at room temperature for 15 min. Finally, a flow cytometer was applied to determine the apoptosis of HUVECs.

### Statistical analysis

Statistical significance was determined using one-way analysis of variance (ANOVA) and Tukey’s test. Statistical analysis was performed using GraphPad Prism software (version 7.0, La Jolla, CA, USA). All statistical data was presented as mean ± standard deviation. A P value of less than 0.05 was regarded as statistically significant.

### Ethics approval

All animal procedures were approved by the Ethics Committee of Xining First People’s Hospital. National Institutes of Health Guide for the Care and Use of Laboratory Animals was followed strictly.

### Availability of data and materials

The datasets used and/or analyzed during the current study are available from the corresponding author on reasonable request.
